# Obstructive sleep apnea and anatomical structures of the nasomaxillary complex in adolescents

**DOI:** 10.1371/journal.pone.0272262

**Published:** 2022-08-04

**Authors:** Jeong-Hyun Kang, Hyun Jun Kim, Seung Il Song

**Affiliations:** 1 Clinic of Oral Medicine and Orofacial Pain, Institute of Oral Health Science, Ajou University School of Medicine, Suwon, Gyeonggi-do, Korea (ROK); 2 Department of Otolaryngology, School of Medicine, Ajou University, Suwon, Gyeonggi-do, Korea (ROK); 3 Department of Oral and Maxillofacial Surgery, Institute of Oral Health Science, Ajou University School of Medicine, Suwon, Gyeonggi-do, Korea (ROK); University of Catania, ITALY

## Abstract

The aim of the present study was to reveal the associations between skeletal and soft tissue features of the nasomaxillary complex and development and severity of obstructive sleep apnea (OSA) in adolescents. A total of 100 adolescents (mean age, 14.9 ± 1.4 years; age range, 13–17 years) were enrolled. All participants underwent full-night polysomnography and had an assessment of size and position of the tongue, tonsillar size, body mass index (BMI), and circumference of the waist, neck, and hip. The skeletal features of the nasomaxillary complex, including the zygomatic arch width, nasal cavity width, nasal base width, intercanine width, intermolar width, maxillary dental arch length, palatal vault angle, palatal depth, and SNA were measured on the three-dimensional images constructed with computed tomography data. Participants with an apnea and hypopnea index (AHI) of lower than 5 (AHI ≤ 5) were classified as control and participants while those with an AHI of greater than 5 were classified as OSA group. Each variable with a significant outcome in the independent T-test and age and sex factors were integrated into the multivariate linear regression and the dependent variable was AHI. There were significant differences in the BMI and hip circumference between two groups. The width of nasal base, palatal vault angle and SNA also showed significant differences between groups. The results from multivariate linear regression demonstrated that the BMI, width of the nasal base, and SNA showed significant contributions to the severity of OSA in adolescents. The features of the nasomaxillary complex seemed to have significant influences on development and severity of OSA.

## Introduction

Obstructive sleep apnea (OSA) is characterized by repetitive complete or partial collapse of the upper airway during sleep, which causes a cessation or reduction in the airflow [1]. A number of studies have pointed out the associations between OSA and compromised cardiovascular and neurobehavioral functions in both adults and children [2–7]. The early detection of risk factors and prompt management of them are important because the severity and duration of OSA increases, so do the clinical consequences.

Although obesity is the main risk factor, the facial profiles and soft tissue features of the upper airway have been regarded as other anatomical risk factors for OSA. Enlarged tonsils may have a role in the development of OSA, especially in pediatrics [8, 9]. The nasomaxillary complex, which is a structure distinguished from the craniofacial complex based on its developmental origin, is the initial part of the upper airway [10]. The relationships between the morphological and anatomical characteristics of the nasomaxillary complex and pharyngeal dimensions have been discussed [11]. Many attempts, including palatal expansion, maxillary protraction, and adenotonsillectomy have been tried to correct enlarged soft tissue and the constricted maxilla and palate and finally enlarge the upper airway dimensions and volumes. These trials have positive impacts on increasing the volume of the upper airway and improving sleep quality [12–18]. Generally, adenotonsillectomy has been regarded as the first-line treatment option for OSA in pediatric patients [19], and the therapeutic effect of palatal expansion and maxillary protraction seem to be maximized in patients around the pubertal growth spurt [11, 20]. Those results might imply that choosing the proper moment for growth stage for the correction of the abnormal nasomaxillary complex and soft tissue structures is important for the prevention and treatment of OSA in the long term. Therefore, clarifying relationships between the skeletal and soft tissue features of the nasomaxillary complex and the severity of OSA in children and adolescents who have not completed their growth is important because certain treatment modalities should be performed at proper growth stages for maximized treatment efficacy.

Adolescence is a unique stage of life during individuals undergo biopsychological transitions from childhood to adulthood which is accompanied by an alteration in growth acceleration, pubertal development, sex and growth hormonal levels, and psychological maturation [21]. The prevalence of OSA in adolescents was estimated to be up to 1.9% [22]. The generation-specific pathophysiology and risk factors for OSA in adolescents have not been clearly revealed and the exact diagnostic criteria for OSA in adolescents also have not been established yet [23]. Previous studies, which tried to reveal relationships among craniofacial anatomical features and OSA in pediatrics, adopted broad age spans and sparse studies focused mainly on adolescents. Therefore, the aim of the present study was to reveal the comprehensive associations between the skeletal and soft tissue features of the nasomaxillary complex and the development and severity of OSA in adolescents.

## Materials and methods

### Participants

This was a cross-sectional study, which was conducted using the clinical and radiographic records and polysomnography (PSG) data of 100 adolescents (male/female, 76/23; mean age, 14.9 ± 1.4 years; age range, 13–17 years) who had been referred to the Sleep Center of Ajou University Hospital, Suwon, Korea, owing to snoring and/or subjective sleep disturbance at night.

The World Health Organization defines an adolescent as anyone between 10 and 19 years of age [24]. To assess dental and occlusal relationships using computed tomography (CT), adolescents with mixed dentition (aged 10–12 years) were excluded. One study suggested the approximate age at which facial growth cessation occurred was during 18–22 years [25]. Therefore, adolescents with completed facial growth (aged over 18 years) were also excluded. Adolescents with craniofacial anomalies, including cleft lip and palate, neurodegenerative disorders, and a history of previous treatment which could influence the dimension of the airway, including adenotonsillectomy and orthodontic treatment, were excluded. All participants had an assessment of size and position of the tongue, tonsillar size, height, weight, body mass index (BMI), and circumference of the waist, neck, and hip. The research protocol was approved by the Institutional Review Board of the university hospital (AJIRB-MED-MDB-18-127). The Institutional Review Board committee approved a request to waive the documentation of informed consent due to the retrospective design of the study.

### Polysomnography

All participants underwent a full overnight in-laboratory PSG (Embla N 7000, ResMed, USA). Total sleep time, sleep latency, sleep efficiency, rapid eye movement (REM) latency, apnea-hypopnea index (AHI), respiratory disturbance index (RDI), respiratory effort related arousal (RERA), and oxygen desaturation index (ODI) were assessed. Determination of apnea was conducted if both of the following were met: 1) There is a drop on the peak signal excursion by more than 90% of pre-event baseline using an oronasal thermal sensor; 2) The duration of the more than 90% drop in sensor signal is more than 10 seconds. The hypopnea was defined if all of the following were met: 1) The peak signal excursion drops by more than 30% of pre-event baseline using nasal pressure.; 2) The duration of the more than 30% drop in signal excursion is more than 10 seconds; 3) There is more than 3% oxygen desaturation from pre-event baseline or the event is associated with an arousal [26]. Total arousal index, the number of arousals per hours and relative snoring time which defined as percentage of snoring time of total sleep time were also determined.

### Diagnosis of OSA

The diagnostic criteria of PSG for adolescents were controversial, and the American Association of Sleep Medicine (AASM) suggested that adolescents could be scored using either pediatric or adult criteria [26]. However, several studies mentioned that adolescents might be more similar to adults in terms of their risk factors for OSA [22, 23, 27], so we adopted adult scoring system for diagnosis of OSA. OSA was determined on the basis of the definition per Center for Medicare and Medicaid Services [28]. The diagnosis requires the observed apnea coupled with an AHI of higher than five. The AHI was calculated as the sum of obstructive and mixed apneas and hypopneas per hour of sleep as defined by the AASM scoring manual [26, 28]. Participants with AHIs of less or equal to than 5 (AHI ≤ 5) were classified as control and those with AHIs of greater than or equal to 5 were classified as the OSA group [29].

### Clinical and radiographic parameters

All participants underwent an assessment of the size and position of the tongue, tonsillar size, height, weight, and circumference of the waist, neck, and hip. The modified Mallampati’s score was used to assess the size and position of the tongue [30] and tonsillar size was evaluated using a grading system proposed in a previous report [31].

**Fig 1:**
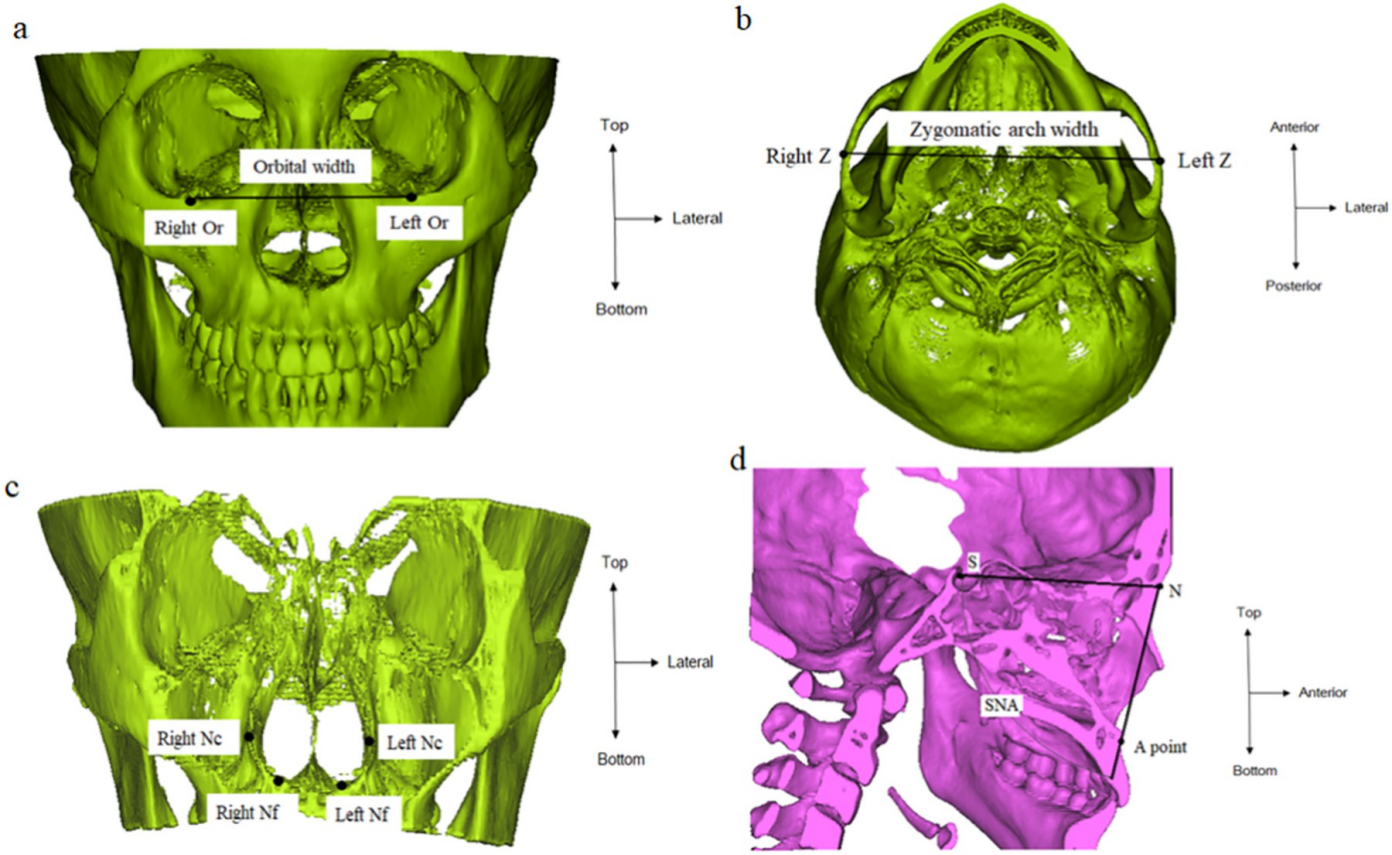
(A) Orbital width (B) Zygomatic width (C) Nasal cavity width and nasal base width (D) SNA, the angle between the Sella-Nasion line and the Nasion-A point line. Or, orbitale; Z, zygomatic process; Nc, the lateral-most points of the nasal cavity; Nf, the junction of the palatal cortical alveolar bone and cortical nasal bone.

**Fig 2:**
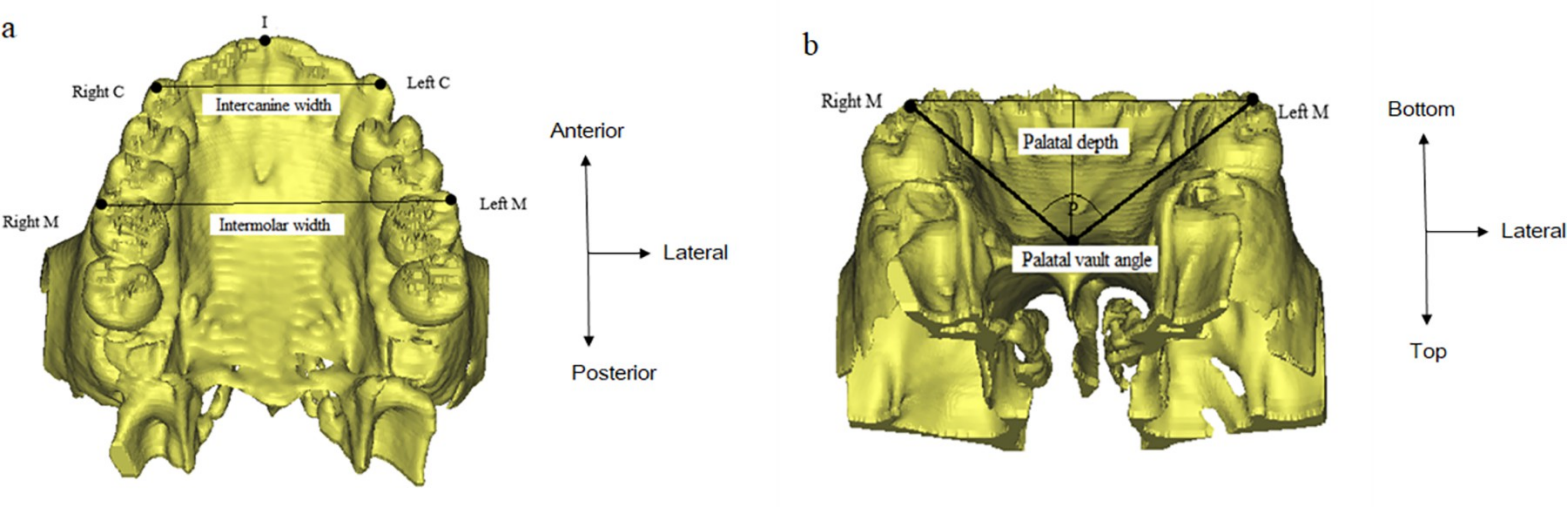
(A) Interdental width (B) Palatal vault angle and palatal depth. C, canine; M, mesiobuccal cusp of the maxillary first molar.

All participants underwent the upper airway CT for screening for skeletal abnormality and soft tissue swelling. The three-dimensional images of the upper airway and nasomaxillary complex were constructed with CT data, using Mimics® software (Materialise, Leuven, Belgium). The following parameters were measured: orbital width, which is defined as the distance between the orbitale (Or) from both sides; zygomatic arch width, indicating the transverse width between the points on the most lateral part of the zygomatic arch on the right and left (Z); nasal cavity width, defined as the transverse width between the points on the most lateral part of each nasal cavity (Nc); nasal base width, indicating the distance between the junction of the palatal cortical alveolar bone and the cortical nasal bone (Nf); SNA, defined as an angle made by the sella (S), nasion (N) and the A point (A) (Fig 1A–1D). Intercanine and intermolar widths were defined as the distances between the right and left canine tips (C)/mesiobuccal cusp tips (M) of the canines and first molars, respectively (Fig 2A). The maxillary arch length was defined as the shortest distance from the tip of the incisor (I) to the line connecting the mesiobuccal cusps (M) of both sides of the maxillary first molars (Fig 2A). Arch length ratio was calculated as the ratio between the intermolar distance and the arch length. The palatal contour was analyzed using the palatal depth and palatal vault angle. The palatal depth was defined as the shortest distance from the deepest part of the hard palate (P) to a line connecting the occlusal surfaces of the mesiobuccal cusps of the first molars. The palatal vault angle indicated the angulation of the intersection lines of the mesiobuccal cusps of both sides of the maxillary first molars to the deepest part of the palatal vault (Fig 2B). To assess inter-examiner reliability, two examiners measured parameters on 30 randomly selected CT images and data from each examiner were compared (inter-examiner) using intraclass correlation coefficients (ICC) to assess the reliability. One observer (KJH) repeated the process after 2 weeks (intra-examiner) and data were compared using ICC. No statistically significant differences were observed.

### Statistical analysis

Sample size calculations were done based on a two-sided type I error of 5% and a power of 80%. The proportion between control and OSA group was based on previous report which demonstrated that prevalence of OSA in habitual snoring adolescents were about 21.2% [32]. Power analysis indicated that a total sample size of 100 participants including 21 OSA patients and 79 controls who involved in this study in the T test would provide a statistical power of 81.6 at a 0.05 significance level with an effect size of 0.4. The effect size was calculated by dividing the differences in group means by the standard deviation of the pooled data of SNA. The normality of data distribution was affirmed using the Shapiro-Wilk normality test, to adopt parametric tests for statistics. The primary outcomes were differences of the parameter related with demographic and craniofacial features and soft tissue characteristics in two groups and secondary outcomes were assessment of the confounding factors to OSA severity. The clinical and radiographic parameters and results from PSG were compared using the independent t-test and Chi-square test for continuous and categorical variables, respectively. Each variable with a significant outcome in the independent T-test was integrated into the multivariate linear regression to identify whether these variables as a whole affected the AHI via multivariate linear regression analysis.

## Results

### Demographic characteristics and results of PSG

Among 100 participants, 79 were classified into the control group and 21 into the OSA group. No significant differences were observed in terms of age and sex distribution between the two groups. Among the anthropometric variables, BMI and hip circumference showed significant differences between the groups (Table 1).

**Table 1:** Comparison of clinical characteristics related to the development of OSA.

	Control (n = 79)	OSA (n = 21)	*P* value
Age	14.8 ± 1.3	15.2 ± 1.5	0.240
Sex (Male/Female) †	59/20	18/3	0.286
BMI	21.9 ± 3.4	26.6 ± 5.8	< 0.001**
Neck circumstance (cm)	36.4 ± 9.3	36.3 ± 3.6	0.955
Waist circumstance (cm)	79.7 ± 15.3	90.1 ± 16.3	0.007*
Hip circumstance (cm)	86.7 ± 18.2	100.9 ± 12.3	< 0.001**
Modified Mallampati score†	2 (2–4)	3 (2–4)	0.290
Tonsillar size†	2 (1–3)	2 (2–3)	0.438
Orbital width (cm)	72.1 ± 4.6	71.7 ± 3.2	0.760
Zygomatic arch width (mm)	132.8 ± 5.8	131.1 ± 5.8	0.295
Width of nasal cavity (mm)	31.9 ± 2.3	33.2 ± 1.9	0.054
Width of nasal base (mm)	20.0 ± 1.7	21.3 ± 4.1	0.039*
Intercanine width (mm)	38.3 ± 1.8	38.3 ± 1.3	0.962
Intermolar width (mm)	55.4 ± 5.1	55.0 ± 2.6	0.684
Maxillary arch length (mm)	26.6 ± 3.0	26.1 ± 3.6	0.568
Arch ratio	2.11 ± 0.30	2.14 ± 0.33	0.731
Palatal vault angle (degree)	119.7 ± 9.7	112.9 ± 6.3	0.010*
Palatal depth (mm)	9.40 ± 2.31	10.9 ± 2.43	0.065
SNA (degree)	80.5 ± 3.9	78.7 ± 2.2	0.018*

BMI, body mass index; OSA, obstructive sleep apnea

Descriptive values are shown as mean ± SD or median (25th– 75^th^ percentile).

Data obtained from independent T-test.

^†^Data obtained from Chi-square test.

* P < 0.05

** *P* < 0.001 by independent T test and Chi square test.

The size and position of the tongue and tonsillar size seemed not to contribute significantly to the development of OSA. The variables related to orbital and zygomatic arch width seemed to have little association with the occurrence of OSA. Similarly, dental variables, including intercanine width, intermolar width, maxillary arch length, arch ratio, and palatal depth did not show significant relationships with the occurrence of OSA. On the other hand, the variables associated with the volume of the nasal cavity such as the width of the nasal base and palatal vault angle, and variables representing the anterior-posterior relationship of the cranium and maxilla such as the SNA showed significant differences (Table 1).

The total sleep time, sleep latency, sleep efficiency, REM latency, and RERA did not show significant differences. On the other hand, AHI, supine AHI, RDI, ODI, total arousal index, and relative snoring time showed significant differences between the two groups (Table 2).

**Table 2:** Polysomnography results of the participants.

	Control (n = 79)	OSA (n = 21)	*P* value
Total sleep time (minute)	395.0 ± 54.5	373.8 ± 99.4	0.747
Sleep latency (minute)	14.5 ± 22.2	16.4 ± 19.0	0.361
Sleep efficiency (%)	86.2 ± 11.7	80.9 ± 20.7	0.133
REM latency (minute)	147.0 ± 67.0	131.6 ± 83.0	0.361
AHI	1.37 ± 1.25	15.1 ± 15.0	< 0.001**
Supine AHI	2.02 ± 3.29	24.8 ± 27.2	< 0.001**
RDI	5.39 ± 3.87	20.5 ± 15.7	< 0.001**
RERA	4.03 ± 3.43	5.41 ± 3.02	0.081
ODI	1.21 ± 1.19	13.3 ± 13.0	< 0.001**
Total arousal index	12.8 ± 5.4	20.2 ± 9.5	< 0.001**
Relative snoring time (%)	15.0 ± 16.0	31.2 ± 20.4	< 0.001**

AHI, apnea-hypopnea index; RDI, respiratory disturbance index; RERA, respiratory effort-related arousal; ODI, oxygen desaturation index

Descriptive values are shown as mean ± SD or median.

Data obtained from independent T-test.

* P < 0.05

** *P* < 0.001 by independent T test.

### Associations among anthropometric parameters, anatomical structures of the nasomaxillary complex, and AHI

The variable with a significant outcome in the independent T-test including BMI, hip circumference, width of the nasal base, palatal vault angle, and SNA and age and sex factors which were known as critical factors for incidence of OSA were integrated into the multivariate linear regression in order to identify whether these variables as a whole affected the severity of OSA. The outcome variable was AHI. The results from multivariate linear regression demonstrated that BMI, width of nasal base, and SNA contributed significantly to the AHI scores in adolescents (Table 3).

**Table 3:** Clinical and radiographic parameters as predictors of the AHI in the multivariate analysis.

	Main effect full model (R^2^ = 0.205)				
	unadjusted		standardized	t	*P* value
	β	S.E	β		
Age	-0.965	0.778	-0.132	-1.240	0.219
Male sex	4.088	2.424	0.181	1.686	0.096
Female sex	Reference				
BMI	0.874	0.288	0.398	3.039	0.003*
Hip circumferences	-0.024	0.074	-0.043	-0.327	0.745
Width of nasal base	1.422	0.636	0.245	2.234	0.030*
Palatal vault angle	-0.058	0.123	-0.053	-0.471	0.639
SNA	-0.821	0.304	-0.303	-2.697	0.005*

AHI, apnea-hypopnea index; S.E, standard error; BMI, body mass index

Data obtained from the multivariate linear regression.

* P < 0.05

** *P* < 0.001 by the multivariate linear regression.

## Discussion

During the last decades, the significant therapeutic effects of many treatment modalities which tried to correct abnormal anatomical structures of the naxomaxillary complex on the increased volume of the upper airway and sleep quality have been proposed and therapeutic effects of some of those modalities are maximized during pubertal growth spurt [12–18]. However, the generation-specific pathophysiology and risk factors associated with structures of the nasomaxillary complex of OSA in adolescent has not been elucidated, so far. The purpose of the present study was to clarify comprehensive associations between skeletal and soft tissue features of nasomaxillary complex and the development and severity of OSA in adolescents. The novel finding of the present study was that the characteristics of the nasomaxillary complex alone without the mandibular component could have a critical role in the development and severity of OSA. Various aspects of the craniofacial features associated with OSA have been investigated in previous studies, and the majority of these studies indicated hypoplastic facial profiles with retruded mandibles as major contributing factors of OSA [33–35]. Furthermore, other previous studies have suggested risk assessment models for OSA using features of the craniofacial structures, and these studies generally included variables related to both maxillary and mandibular components [36–38]. One study suggested the importance of horizontal maxilla-skull base relationships in the development of OSA, but the suggested model in this study also included mandibular variables [39]. Considering the unique maxillary features of OSA patients such as a constricted maxillary dental arch and a retruded maxilla, the important role of the nasomaxillary complex in the development of OSA could be assumed. An increase in the volume of the oral cavity owing to a wider lateral maxillary dimension may result in the anterior displacement of the tongue, and a protrusive maxilla may be associated with increased nasopharyngeal airway dimensions [12–14, 16–18, 40, 41]. Therefore, the nasomaxillary complex itself might have a sufficient role in the development of OSA.

There are some risk factors for OSA need to be assessed differently for adults and children. For example, the contribution of adenotonsillar hypertrophy may be different in pediatric and adolescent OSA. The tonsillar size showed positive correlations with AHI in toddlers and preschoolers but not in adolescents [42]. Our results supported this idea that there were no significant differences in tonsillar size between participants with OSA and controls. The tonsil reaches its greatest size between the ages of 7 years and 10 years, and it has gradually decreased since then. Thus, the effect of tonsillar size on the development of OSA in adolescents seems to be little [43]. However, tonsillar hypertrophy could influence the growth of the maxilla and palate in the long term. A large adenoid could obstruct nasal breathing and may lead to mouth breathing and the lower displacement of the tongue [44]. The compromised balance between forces from the cheeks and tongue, owing to the lower displacement of the tongue, may interrupt lateral maxillary growth [44]. Therefore, although adenotonsillar hypertrophy itself may not have a critical influence on the pathophysiology of OSA in adolescents, it might influence the growth of the nasomaxillary complex in the long term in adolescent OSA patients.

The modified Mallampati score has been regarded as a simple and valid method of assessing relationships between the tongue, soft palate, and oral cavity [30]. This has been considered a reliable predictor of OSA, but the above results showed a lack of significant relationships between the development of OSA and the modified Mallampati score in adolescents. The modified Mallampati score has been shown to have correlations with severity of the OSA in adults [45], but in pediatrics, the results remain controversial [46, 47]. In addition, total soft tissue volumes, including the tongue, soft palate, and fat pad in the pharyngeal wall showed prominent influences on the development of OSA in adult patients [48] but their roles were not obvious in adolescent OSA patients [49]. These findings suggest that the critical contributing factor is not a single anatomical abnormality but rather the combination of deficiencies involving the nasomaxillary complex, position of the mandible, and features of the soft tissues especially in individuals with incomplete facial growth.

The results from multivariate linear regression showed that BMI, the width of the nasal base, and SNA seemed to have significant influences on the severity of OSA. The impacts of obesity on development and severity of OSA have been well-known [50, 51]. Maxillary protraction may lead to increased nasopharyngeal and upper oropharyngeal dimensions and an improvement in sleep apnea [13, 16]. The positive relationships between SNA value which reflects the anterior-posterior relationship between cranial base and maxilla and AHI could be understood in this manner. The effects of palatal or maxillary expansion on an increase in the volume of the nasal cavity in both pediatric and adult OSA patients have been generally accepted [40, 41], but their influence on the improvement of OSA remains controversial [52]. Interestingly, several studies showed increased volumes of the lower pharyngeal airway after maxillary expansion [13, 16]. These studies suggested that the larger space provided by the correction of transverse maxillary deficiency would result in increased lower pharyngeal dimensions owing to the anterior displacement of the tongue. Finally, this may result in decreased AHI [12, 14, 17, 18]. Those prospective studies which tried to correct narrow palatal width and nasal base seemed to have significant influences on improvement of OSA, but the results from the present cross-sectional study showed inconsistent results. The results from independent T-test demonstrated that larger nasal base width was detected in healthy adolescents compared to those in adolescents with OSA, but results from multivariate regression analysis showed that positive correlations between nasal base width and AHI. The soft tissue features related with nasal obstruction such as inferior turbinate hypertrophy and septal deviation seemed to play critical role in severity of OSA [53], but influences of skeletal factors remains to be obscure. Hence, the therapeutic effects of palatal expansion or maxillary protrusion would not be the sole result from the increased skeletal width of the nasal cavity and base, but the combined results from soft tissue characteristics including inferior turbinate and nasal septum and tongue position.

Many previous studies which attempt to reveal the associations between craniomaxillofacial features and severity of OSA have focused on the upper airway volume or cross-sectional area of pharynx [12, 18, 34, 35, 54–56]. However, the discrepancies among those factors that the attempts for correction of skeletal features and enlargement of the airway dimensions such as palatal expansion would not result in improvement of sleep quality were also reported [57]. Another report suggested the importance of harmonized craniofacial skeletal and soft tissue structures on maintaining pharyngeal tension and preventing airway collapse [58]. Especially in adolescents with uncomplete facial growth, the imbalanced growth between skeletal and soft tissue would be more critically affect the development and severity of OSA.

There are several limitations to this study. Firstly, the present study simply defined the adolescents as individuals aged between 13–17 and did not consider the endocrinological factors. Secondly, because the present study was a hospital-based study, the participants were recruited from a tertiary medical center and not from the community. Thirdly, because only CT data were utilized in the study, limited information about soft tissue size measurement was provided. Finally, owing to the relatively small sample size, especially in the OSA group, the power of statistical significance is inevitably compromised. Furthermore, owing to the small number of female participants, this study could provide limited information about the sex contribution in the development of OSA in adolescents. Future studies with larger samples of participants recruited from the community and analysis about hormonal levels and soft tissue size measurement should be conducted to further elucidation of the risk factors for OSA in adolescents.

Adolescence is different from childhood and adulthood, and the diagnosis and management of OSA in adolescents should differ from that in pediatrics or adults. A comprehensive understanding of the anatomical elements of the upper airway in terms of skeletal and pharyngeal growth rather than the quantification of anatomical abnormalities is essential for the proper management of OSA in adolescents.
